# Impacts of *GRIN3A*, *GRM6* and *TPH2* genetic polymorphisms on quality of life in methadone maintenance therapy population

**DOI:** 10.1371/journal.pone.0201408

**Published:** 2018-07-30

**Authors:** Ruey-Yun Wang, Hsiu-Ju Chen, Chieh-Liang Huang, Jiun-Yi Wang, Tsui-Er Lee, Hsiang-Yen Lee, Chin-Chuan Hung

**Affiliations:** 1 Department of Public Health, China Medical University, Taichung, Taiwan, R.O.C; 2 Brain Disease Research Center, China Medical University Hospital, Taichung, Taiwan, R.O.C; 3 Department of Pharmacy, College of Pharmacy, China Medical University, Taiwan, R.O.C; 4 Center for Drug Abuse and Addiction, China Medical University Hospital, Taichung, Taiwan, R.O.C; 5 College of Medicine, China Medical University, Taichung, Taiwan, R.O.C; 6 Department of Healthcare Administration, Asia University, Wufeng, Taichung, Taiwan, R.O.C; 7 Office of Physical Education, Asia University, Taichung, Taiwan, R.O.C; 8 Department of Internal Medicine, Taipei Medical University Hospital, Xinyi District, Taipei City, Taiwan, R.O.C; 9 Department of Pharmacy, China Medical University Hospital, Taichung, Taiwan, R.O.C; Peking University, Institute of Mental Health, CHINA

## Abstract

Opioid addiction is a major public health issue worldwide. Methadone maintenance treatment (MMT) is used to detoxify users of illicit opiates, but drug relapse is common and associated with poor quality of life (QoL). This study investigated the associations between the *GRIN3A*, *GRM6*, and *TPH2* genetic variants and QoL in the MMT population. A total of 319 participants were included in the study, and genotyping of *GRIN3A*, *GRM6*, and *TPH2* genes was performed using the Sequenom iPLEX. Associations between genotypes and the domains of QoL were examined through posthoc analysis with LSMEANS syntax using SAS 9.1.3. The single nucleotide polymorphisms rs9325202 and rs1487275 in the *TPH2* gene were significantly associated with the QoL domain of physical functioning. The least absolute shrinkage and selection operator regression model revealed that the risk allele rs1487275-G was significantly correlated with the domain of physical functioning when clinical characteristics were considered as covariates. The results of the present study illuminate the importance of the genetic basis of QoL in the MMT population, and suggest that genotypes should be considered as a potential QoL indicator.

## Introduction

The health burden associated with heroin use is well documented, but the quality of life (QoL) of patients undergoing methadone maintenance treatment (MMT) to overcome a drug habit has been less well studied. In medical research, QoL is a key index for evaluating health status and identifying the principal problems faced by people in various phases of life [1]. Opioid addiction is a major public health issue worldwide [2]. MMT reduces illicit opiate abuse, decreases the incidence of risky behavior that can result in the transmission of the human immunodeficiency virus (HIV), and extends the lives of injection drug users; however, relapse is common among those participating in MMT programs [3]. One study reported that poor QoL was associated with drug relapse among opioid users in an MMT program [4]. However, few studies have identified the major factors associated with QoL in the MMT population.

Evaluating QoL on the basis of physical, mental, and social well-being may provide clinicians with a holistic perspective of an individual’s condition [5, 6]. Sex, education, occupation, income, and HIV status have been reported to be key factors potentially related to QoL scores in the MMT population [7–9], but research has not yet examined whether genetic factors are associated with QoL scores or are determinants of individual QoL items. Several studies have suggested that numerous genetic variants may play influential roles in the etiology of heroin addiction [10–12]. The *N*-methyl-D-aspartate (NMDA) receptor was revealed to be related to the development of neuropsychiatric disorders such as drug addiction [13]. Inhibition of NMDA neurotransmission has been determined to not only block the development of morphine dependence but also reduce the occurrence of withdrawal symptoms [14, 15]. The components of the NMDA receptor are two NR1 subunits and two subunits from the NR2 and NR3 families. The NR3 subunits have two subtypes, NR3A and NR3B, and have been associated with a neuroprotective function and cocaine-induced addiction [16–19]. The NR3A subtype is encoded by *GRIN3A*, which is located on chromosome 9q31.1 with proximity to 170 kb. NR3A expression has been reported to be correlated with heroin withdrawal symptoms and cocaine-induced glutamatergic transmission [19, 20]. In addition, the genetic variants in NR3 subunits may influence human brain function [19, 21, 22]. In light of these findings, additional studies that focus on whether the QoL of the MMT population is affected by the genetic variants in NR3A and whether the genetic variants are associated with drug relapse in opioid users in MMT programs are warranted.

Glutamate receptor metabotropic 6 (*GRM6*) and tryptophan hydroxylase 2 (*TPH2*) have also been reported to be involved in heroin dependence [19, 20, 23, 24]. *GRM6* encodes a glutamate receptor subunit and is related to the pathophysiology of visual function, autism, mood disorders, and addiction [25, 26]. Glutamate homeostasis is critical for mood and addiction behavior, and alterations in the expression or function of glutamate receptors may contribute to opiate addiction [27, 28]. Tryptophan hydroxylase is the rate-limiting enzyme involved in serotonin biosynthesis [29]. Serotonin plays a crucial role in the regulation of multiple aspects of mood and impulsivity [30], and thus the factors that influence serotonin expression may also affect—and possibly result in a deficit of—impulse control [31, 32]. *TPH* has two isoforms, *TPH1* and *TPH2*, which are mainly expressed in the pineal gland and raphe nuclei in the brain, respectively [33–35]. The genetic variants in *TPH1* and *TPH2* have been reported to be associated with alcoholism, nicotine dependence, and heroin addiction [24, 36–38]. Therefore, the genetic variants that influence the function or expression of glutamate receptors and the biosynthesis of serotonin could be associated with drug relapse in the MMT population. However, no reports have been published on correlations between the genetic variants in *GRM6*, *TPH1*, and *TPH2* and QoL or drug relapse in the MMT population.

The objective of the present study was to explore the integrative effects of NMDA and glutamatergic and serotoninergic neurotransmission-related genes on QoL in the MMT population. The Medical Outcome Studies 36-Item Short-Form Health Survey, a standard questionnaire used to evaluate QoL, was employed in the present study to assess clinical characteristics, social status, and multiple genetic variants in the study participants in order to investigate possible correlations between the genetic variants and QoL in the MMT population.

## Materials and methods

### Subjects

The study protocol was reviewed and approved by the institutional review board of China Medical University Hospital (DMR98-IRB-166) and was in compliance with the Declaration of Helsinki.

Han Chinese participants were enrolled with the following inclusion criteria: (1) with heroin dependence and under methadone maintenance treatment in China Medical University Hospital; (2) signed the written informed consent; (3) within normal EKG; (4) not using concurrent medications which may affect methadone metabolism. The following clinical information was recorded for each patient: gender, weight (kg), height (cm), liver function, comorbidities and the daily dose of methadone. The Medical Outcome Studies 36-item Short-Form Health Survey (SF-36) was used to evaluate the QoL of the participants either by a trained nurse or self-reported. There are 36 items in the SF-36 and these items are divided into eight domains of physical health (Physical Function, Role-Physical, Bodily Pain and General Health) and mental health (Vitality, Social Functioning, Role-Emotional and Mental Health) (S1 Table). The score of each item would be calculated according to the manual.

### Candidate variants selection and genotyping

It is known that several variants in *GRIN3A*, *GRM6* and *TPH2* genes may be associated with expression and function of these enzymes or receptors. Among these variants, it has been shown that rs7030238, rs1983812, rs942142, rs10512285, and rs3983721 in *GRIN3A* gene, rs17078853, rs2071247, rs17078877, rs11746675, rs2067011 in *GRM6* gene, and rs2129575, rs1386493, rs2171363, rs7305115, rs10506645, rs4760820, rs9325202 and rs1487275 in *TPH2* gene were the eighteen single-nucleotide polymorphisms (SNPs) with minor allele frequency more than 5% in Asia population according to NCBI database. Hence, these eighteen variants were selected for investigation in the present study.

DNA was extracted from 3–10 ml of whole blood by using the QIAamp DNA Blood mini Kit (Qiagen, Valencia, CA, USA) according to the manufacturer’s protocol. The genotyping procedure was performed at the National Center for Genome Medicine, Taiwan by the Sequenom iPLEX matrix-assisted laser desorption/ionization time-of-flight mass-spectrometry technology. We randomly selected 20 duplicates samples for quality control and the concordance rate was >0.99 for all SNPs assayed.

### Statistical analysis

Each SNP genotype frequency distribution was examined for Hardy-Weinberg equilibrium (HWE) by using the chi-square one-degree of freedom goodness-of-fit test. Demographic characteristics and clinical parameters were evaluated with a chi-squared contingency table for categorical variables and Wilcoxon's U test for continuous variables among the groups. We calculated Cohen’s *d* by each group n, mean, and standard deviation at given two-tailed alpha = 0.05 for power level = 0.80 (80%) in SNPs reached significant difference between domain of Physical functioning or domain of Role-Physical of QoL to estimate the sample size.

Linkage disequilibrium (LD) plot was generated by a commonly used bioinformatics software, Haploview (version 4.2), computing pairwise LD statistics for SNPs within a certain distance of each other and analyzing the patterns of SNPs in the present study subjects[39]. Furthermore, we also performed LDlink, a web-based applications designed to interrogate linkage disequilibrium in population groups, to illustrate the LD plots as well[40]. The CHB (Han Chinese in Beijing, China) genotypes data was used to generate pairwise LD plots from Phase 3 (Version 5) of 1000 Genomes Project and variant rs numbers were indexed based on dbSNP build 142 with LDlink. The R^2^ values range from 0 to 1 and higher values indicate higher degree of correlation. The post hoc analysis with LSMEANS syntax was performed in individual SNP and haplotypes for domains of Physical Functioning and Role-Physical[41]. The multiple correction related to multiple SNPs was handled by SAS automatically.

The LASSO (least absolute shrinkage and selection operator) regression method was used for linear model (domains of QoL = demographic characteristics + methadone treatment dose + SNPs). It is a powerful penalty-based method used in predictor selection to avoid model overfitting and a more robust statistical methodology than standard variable selection methods (forward, backward or stepwise). The LASSO model uses PROC GLMSELECT, selection SBC (the Schwarz Bayesian Information Criterion) based on likelihood function and evaluates for all models obtained by deleting an effect from the current model or by adding an effect to this model[42, 43].

The ancestry of population in the present study is Han Chinese, with similar genetic background. Data analyses were conducted using SAS and SAS/Genetics software, version 9.1.3 (SAS Institute, Cary, NC). A two-sided *P*-value <0.05 was considered statistically significant.

## Results

### Subjects

A total of 319 participants (253 men/66 women) were included and further divided into three groups based on their maximum stabilized methadone daily doses: less than 55 mg/day, between 56 and 99 mg/day, and more than 100 mg/day. Due to the fact that the observed methadone dose have a tri-modal distribution, the methadone dose groups was decided according to the distribution of the dosage of included participants. The basic characteristics of participants among the three methadone dosage groups were not significantly different (Table 1).

**Table 1:** Demographic data of included subjects of methadone maintenance therapy.

		Maxdose≦55 mg		56 mg<Maxdose<99 mg		Maxdose≧100 mg		*P*-value
Variable		N	Mean±SD	N	Mean±SD	N	Mean±SD	
Gender								
	Male	77	(30.43%)	119	(47.04%)	57	(22.53%)	0.3726
	Female	22	(33.33%)	25	(37.88%)	19	(28.79%)	
Education level								
	Elementary school or less	5	(23.81%)	12	(57.14%)	4	(19.05%)	0.5707
	Junior high school	48	(33.57%)	65	(45.45%)	30	(20.98%)	
	Senior high school	46	(29.68%)	67	(43.23%)	42	(27.10%)	
Marital status								
	Never-married	55	(30.39%)	78	(43.09%)	48	(26.52%)	0.6488
	Married	24	(35.29%)	31	(45.59%)	13	(19.12%)	
	Divorce	19	(29.69%)	32	(50.00%)	13	(20.31%)	
Age		99	42.23±7.13	144	42.88±7.52	76	40.38±7.15	0.0554
SGOT		93	40.43±38.73	140	42.75±29.25	74	46.89±59.15	0.5967
SGPT		93	48.25±43.48	139	55.02±52.59	74	57.16±74.35	0.5425
rGT		92	45.96±61.84	135	37.46±28.35	74	37.64±51.24	0.3524
BMI		91	22.90±3.15	133	22.85±2.90	72	22.34±2.40	0.4003
Items of SF-36								
Physical Function		92	25.04±5.30	136	24.42±4.71	73	24.34±4.38	0.5560
Role-Physical		91	5.66±1.69	135	5.24±1.48	73	5.26±1.61	0.1142
Bodily Pain		92	9.30±1.76	139	8.85±2.12	73	9.01±1.69	0.2101
General Health		91	14.41±3.38	137	14.19±3.72	72	13.16±3.32	0.0575
Vitality		90	13.70±3.19	136	13.35±2.89	72	13.08±3.26	0.4370
Social Functioning		91	6.60±1.65	138	6.34±1.41	72	6.56±1.48	0.3718
Role-Emotional		91	4.08±1.22	135	3.82±1.16	73	4.07±1.33	0.2105
Mental Health		90	18.17±3.74	136	17.36±3.14	72	17.58±4.21	0.2542

Note: Data in parentheses are shown in percentage.

SGOT: serum glutamic oxaloacetic transaminase; SGPT: serum glutamic-pyruvic transaminase; rGT: r-glutamyl transferase; BMI: body mass index

### The associations between genotypes and domains of QoL

The genotype frequencies of the *GRIN3A*, *GRM6* and *TPH2* polymorphic loci and the score of each item of SF-36 of participants were listed in S2 Table. The genotypic distribution of each genotype was consistent with Hardy-Weinberg equilibrium proportions (S3 Table). The associations between genotypes and domains of QoL were examined by the post hoc analysis with LSMEANS syntax and there were two SNPs, rs9325202 and rs1487275 in *TPH2* gene, significantly associated with domain of Physical Functioning of QoL (Table 2). The sample size power was estimated by the Cohen’s *d* method and in domain of Physical functioning, the rs942142 was genotyped in 119 subjects and the power was calculated at 77.8%. The rs9325202 was genotyped in 249 subjects and the power was calculated at 88%. The rs1487275 was genotyped in 249 subjects and the power was calculated at 88%. In domain of Role-Physical, the rs942142 was genotyped in 120 subjects and the power was calculated at 96.1%. The rs17078853 was genotyped in 251 subjects and the power was calculated at 65.9%. The rs11746675 was genotyped in 247 subjects and the power was calculated less than 25%. Therefore, the powers of comparisons in the domain of Physical functioning reached 80%, while some of the comparisons in the domain of Role-Physical did not.

**Table 2:** Associations between *TPH2*, *GRIN3A*, and *GRM6* genotypes and QoL of participants.

Gene	SNP	Allele	Physical Functioning			P-value	Role-Phusical			P-value
			LSMEANS	Lower CL	Upper CL		LSMEANS	Lower CL	Upper CL	
*GRIN3A*	rs7030238	A vs C	1.01	-2.22	4.25	0.6557	0.28	-0.78	1.34	0.7828
	rs1983812	G vs A	0.38	-2.06	2.82	0.7803	0.73	-0.06	1.52	**0.0459**
	rs942142	C vs A	2.97	-1.34	7.28	0.2368	1.34	-0.02	2.70	0.0520
	rs10512285	G vs A	2.67	-0.82	6.16	0.1454	1.08	-0.08	2.24	0.0605
	rs3983721	C vs T	0.77	-1.32	2.85	0.5851	0.48	-0.21	1.16	0.1773
*GRM6*	rs17078853	T vs G	0.97	-3.61	5.55	0.5025	0.39	-1.09	1.88	**0.0409**
	rs2071247	A vs G	0.63	-1.60	2.87	0.7841	0.27	-0.46	1.00	0.6663
	rs17078877	A vs G	1.03	-3.46	5.51	0.3988	0.40	-1.09	1.89	0.0695
	rs11746675	T vs C	0.57	-1.85	2.99	0.7604	0.19	-0.60	0.98	0.0614
	rs2067011	T vs C	0.39	-2.12	2.90	0.8880	0.18	-0.60	0.97	0.4310
*TPH2*	rs2071247	A vs G	0.63	-1.60	2.87	0.2411	0.27	-0.46	1.00	0.8325
	rs1386493	T vs C	0.28	-3.74	4.31	0.3198	0.70	-0.63	2.04	0.3598
	rs2171363	C vs T	0.62	-1.50	2.74	0.2991	0.11	-0.60	0.83	0.7634
	rs7305115	G vs A	0.62	-1.55	2.79	0.3148	0.22	-0.50	0.94	0.4773
	rs10506645	C vs T	1.98	-0.34	4.30	0.1077	0.35	-0.42	1.12	0.3524
	rs4760820	G vs C	0.51	-7.97	9.00	0.9845	2.13	-0.62	4.88	0.1508
	rs9325202	G vs A*	2.32	0.17	4.46	**0.0260**	0.51	-0.20	1.22	0.1044
	rs1487275	T vs G*	2.64	0.03	5.26	**0.0430**	0.60	-0.26	1.46	0.1593

*p<0.05 denoted statistical significance.

**Fig 1:**
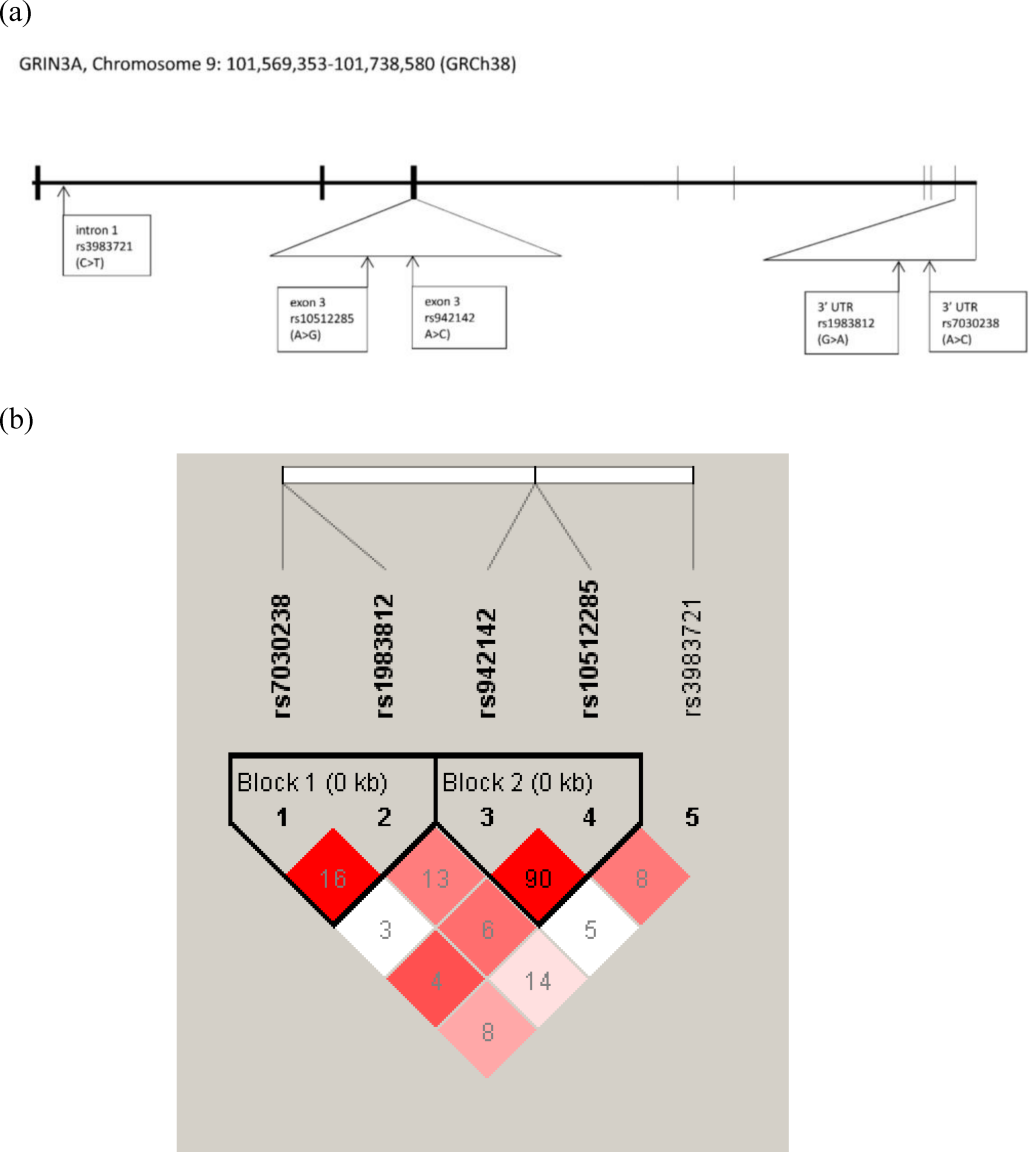
The genomic location and linkage disequilibrium pattern of the *GRIN3A* genetic polymorphisms included in this study. Genomic locations of the genetic polymorphisms on chromosome 9. Haploview 4.2 software was used to estimate the linkage disequilibrium blocks. The R^2^ values were shown in squares; range from 0.03 to 0.90 and higher values indicate higher degree of correlation.

**Fig 2:**
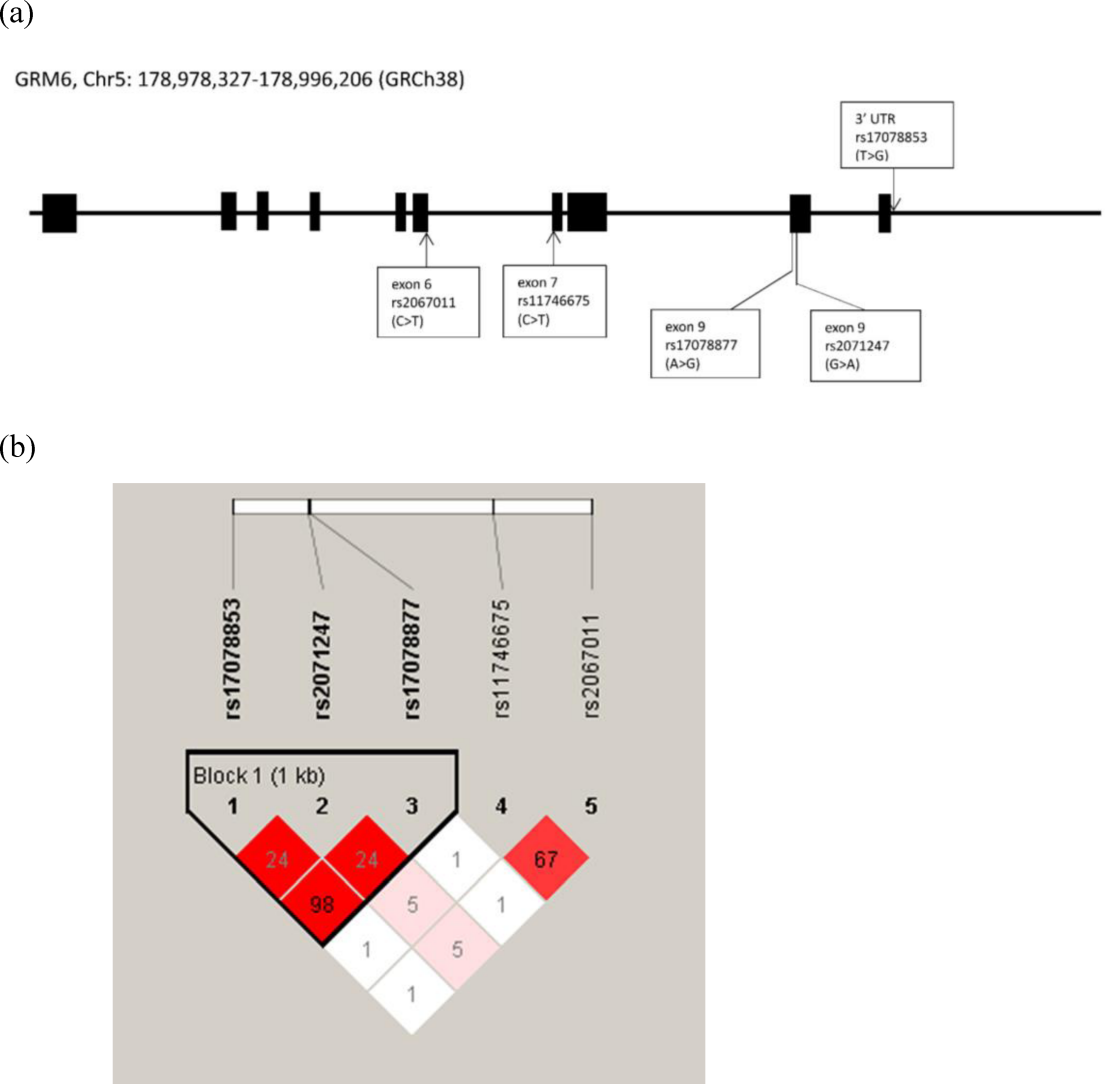
The genomic location and linkage disequilibrium pattern of the *GRM6* genetic polymorphisms included in this study. Genomic locations of the genetic polymorphisms on chromosome 5. Haploview 4.2 software was used to estimate the linkage disequilibrium blocks. The R^2^ values were shown in squares; range from 0.01 to 0.98 and higher values indicate higher degree of correlation.

**Fig 3:**
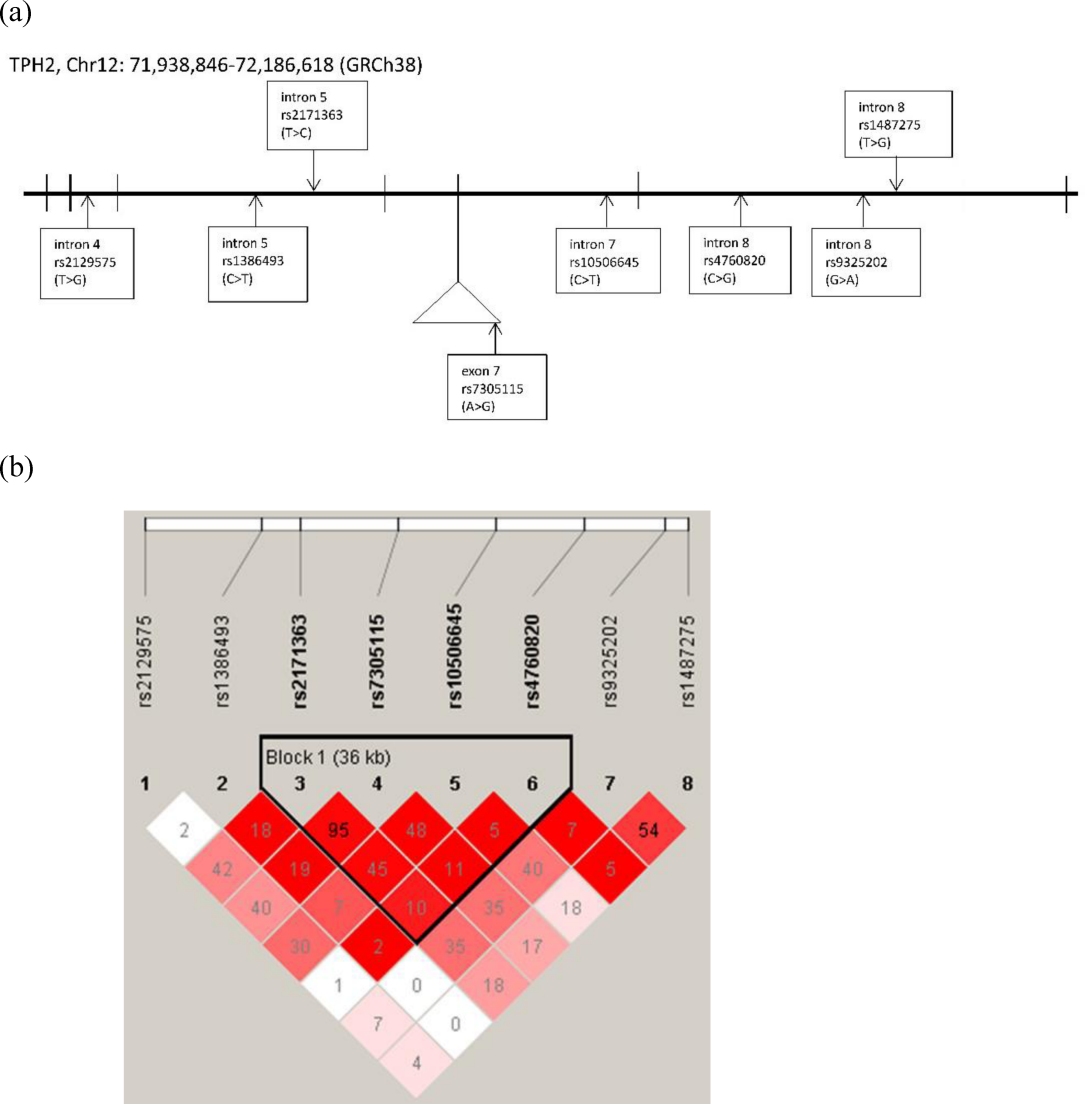
The genomic location and linkage disequilibrium pattern of the *TPH2* genetic polymorphisms included in this study. Genomic locations of the genetic polymorphisms on chromosome 12. Haploview 4.2 software was used to estimate the linkage disequilibrium blocks. The R^2^ values were shown in squares; range from 0 to 0.95 and higher values indicate higher degree of correlation.

The genomic locations and linkage disequilibrium patterns of the *GRIN3A*, *GRM6* and *TPH2* genetic polymorphisms were generated by LDlink in CHB database (S1, S2 and S3 Figs) and by Haploview in the present study subjects (Figs 1, 2 and 3) as well. There are no significant discrepancies between LD structures of CHB population and the study population of the present study. There were two LD blocks (rs7030238 and rs1983812; rs942142 and rs10512285) in *GRIN3A* gene were generated from five SNPs in both CHB database and the present study subjects (Fig 1 and S1 Fig). One LD block (rs17078853, rs2071247 and rs17078877) in *GRM6* gene was generated from five SNPs in both CHB database and present study subjects. In addition, another LD block (rs11746675 and rs2067011) was only shown in CHB database but not in present study subjects (Fig 2 and S2 Fig). There was one LD block (rs2171363, rs7305115, rs10506645, and rs4760820) in *TPH2* gene generated from eight SNPs in the present study subjects, while two LD blocks (rs2171363 and rs7305115, rs9325202 and rs1487275) were generated in CHB database (Fig 3 and S3 Fig). Further haplotype analysis by post hoc analysis with LSMEANS syntax demonstrated that no haplotype in *GRIN3A* or *TPH2* gene was associated with performance of domains of Physical Functioning or Role-Physical (Table 3).

**Table 3:** Associations between *GRIN3A* and *TPH2* haplotypes and QoL of participants.

Gene	haplotype	haplotype	Physical Functioning			Role-Physical		
			LSMEANS	Lower CL	Upper CL	LSMEANS	Lower CL	Upper CL
*GRIN3A*	rs942142-	C-G/C-G vs A-A/C-A	0.83	-5.90	7.56	0.92	-1.30	3.13
	rs10512285	C-G/C-G vs A-A/C-G	2.32	-1.88	6.53	1.15	-0.23	2.53
		C-G/C-G vs A-A/A-A	2.74	-1.29	6.76	1.09	-0.24	2.41
		A-A/C-A vs A-A/C-G	1.49	-4.23	7.21	0.24	-1.64	2.12
		A-A/C-A vs A-A/A-A	1.90	-3.69	7.50	0.17	-1.67	2.01
		A-A/C-G vs A-A/A-A	0.41	-1.50	2.33	0.07	-0.56	0.69
*TPH2*	rs9325202-	G-T/G-T vs A-T/G-T	0.21	-3.94	4.37	-0.51	-1.88	0.86
	rs1487275	G-T/G-T vs A-G/G-T	0.44	-2.35	3.23	0.08	-0.85	1.00
		G-T/G-T vs A-G/A-G	2.00	-2.21	6.22	0.28	-1.10	1.67
		G-T/G-T vs G-G/G-T	2.36	-4.00	8.73	0.23	-1.87	2.32
		G-T/G-T vs A-G/A-T	2.49	-1.95	6.93	0.64	-0.82	2.10
		G-T/G-T vs A-T/A-T	4.25	-13.96	22.46	1.45	-4.55	7.45
		G-T/G-T vs A-G/G-G	5.92	-4.72	16.56	0.78	-2.72	4.29
		A-T/G-T vs G-T/G-T	-0.21	-4.37	3.94	0.51	-0.86	1.88
		A-T/G-T vs A-G/G-T	0.23	-3.86	4.32	0.59	-0.77	1.94
		A-T/G-T vs A-G/A-G	1.79	-3.38	6.96	0.79	-0.91	2.50
		A-T/G-T vs G-G/G-T	2.15	-4.88	9.19	0.74	-1.58	3.05
		A-T/G-T vs A-G/A-T	2.28	-3.08	7.63	1.15	-0.61	2.91
		A-T/G-T vs A-T/A-T	4.04	-14.41	22.49	1.96	-4.12	8.04
		A-T/G-T vs A-G/G-G	5.71	-5.35	16.76	1.29	-2.35	4.93
		A-G/G-T vs G-T/G-T	-0.44	-3.23	2.35	-0.08	-1.00	0.85
		A-G/G-T vs A-T/G-T	-0.23	-4.32	3.86	-0.59	-1.94	0.77
		A-G/G-T vs A-G/A-G	1.56	-2.60	5.72	0.21	-1.16	1.58
		A-G/G-T vs G-G/G-T	1.92	-4.40	8.25	0.15	-1.93	2.24
		A-G/G-T vs A-G/A-T	2.05	-2.34	6.43	0.57	-0.88	2.01
		A-G/G-T vs A-T/A-T	3.81	-14.39	22.01	1.38	-4.62	7.37
		A-G/G-T vs A-G/G-G	5.48	-5.14	16.10	0.71	-2.79	4.21
		A-G/A-G vs G-T/G-T	-2.00	-6.22	2.21	-0.28	-1.67	1.10
		A-G/A-G vs A-T/G-T	-1.79	-6.96	3.38	-0.79	-2.50	0.91
		A-G/A-G vs A-G/G-T	-1.56	-5.72	2.60	-0.21	-1.58	1.16
		A-G/A-G vs G-G/G-T	0.36	-6.71	7.43	-0.06	-2.38	2.27
		A-G/A-G vs A-G/A-T	0.49	-4.92	5.90	0.36	-1.42	2.14
		A-G/A-G vs A-T/A-T	2.25	-16.22	20.72	1.17	-4.92	7.25
		A-G/A-G vs A-G/G-G	3.92	-7.16	15.00	0.50	-3.15	4.15
		G-G/G-T vs G-T/G-T	-2.36	-8.73	4.00	-0.23	-2.32	1.87
		G-G/G-T vs A-T/G-T	-2.15	-9.19	4.88	-0.74	-3.05	1.58
		G-G/G-T vs A-G/G-T	-1.92	-8.25	4.40	-0.15	-2.24	1.93
		G-G/G-T vs A-G/A-G	-0.36	-7.43	6.71	0.06	-2.27	2.38
		G-G/G-T vs A-G/A-T	0.13	-7.08	7.34	0.41	-1.96	2.79
		G-G/G-T vs A-T/A-T	1.89	-17.19	20.96	1.22	-5.06	7.50
		G-G/G-T vs A-G/G-G	3.56	-8.51	15.62	0.56	-3.42	4.53
		A-G/A-T vs G-T/G-T	-2.49	-6.93	1.95	-0.64	-2.10	0.82

### Regression model analysis

**Fig 4:**
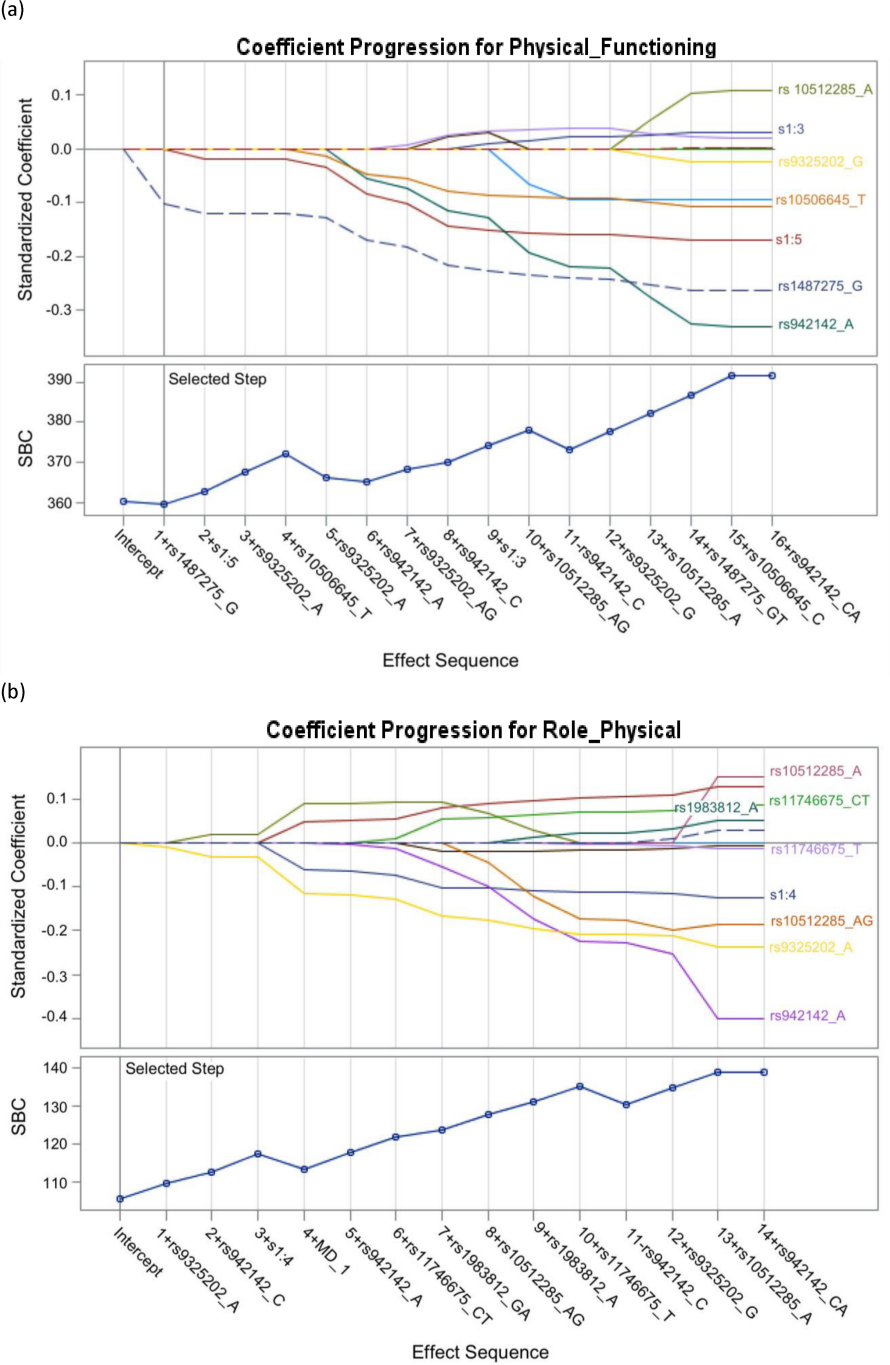
The LASSO regression model. (a) A risk factor rs1487275_G was selected in the LASSO regression model. (b) No risk factor was selected in the LASSO regression model to predict Role-Physical of QoL.

The demographic characteristics, age, categories of methadone treatment dose, and SNPs were independent variables in the LASSO regression model to predict the dependent variable QoL. There was only one factor, a risk allele rs1487275-G, was selected by the LASSO selection method to predict the domain of Physical Functioning (Fig 4A) and the coefficient estimate of rs1487275_G is close to 0 (- 1.347569). There was no variable selected by the LASSO selection method to predict the domain of Role-Physical (Fig 4B).

## Discussion

The present study identified several genetic risk factors associated with the QoL of the MMT population. We found that the scores obtained by patients undergoing MMT in the domain of Physical Functioning were significantly influenced by *TPH2* rs9325202 G>A and rs 1487275 T>G. In addition, the *TPH2* rs1487275-G was identified as risk allele by the LASSO regression model.

Factors associated with the QoL of the MMT population that have been previously reported were mainly focused on social status[7–9]. The QoL of the MMT population has been related to marital status, main source of income, sexual behaviours, HIV infection, and social support[9–12]. After the confounders of age, gender, education, and municipality were controlled for, HIV infection was still significantly related to lower QoL scores in the MMT population[8]. In addition to these socioeconomic factors, biological pathways and neurotransmitters such as serotonergic and glutamatergic pathways have been identified as related to various QoL domains, including pain, fatigue, and emotional and social functioning[44–47]. Since it was proposed in 2004, the hypothesis that QoL has a genetic basis has been supported by several studies[48, 49]. In the present study, we discovered that *TPH2* rs1487275-G was significantly associated with the QoL of the MMT population after adjustment of clinical and socioeconomic factors, supported the hypothesis that QoL has a genetic basis.

Serotonin (5-HT) is a monoamine that functions as a neurotransmitter or peripheral hormone. TPH isoform 2, encoded by *TPH2*, catalyses the rate-limiting step in the synthesis of 5-HT. Genetic polymorphisms of *TPH2* have been demonstrated to be related to altered TPH2 expression in the central nervous system, symptoms of depression, hopelessness, cocaine addiction, and heroin addiction[24, 50, 51]. The influence of TPH2 expression on brain function and susceptibility to depression was supported in the transgenic animal model[52]. Thus, TPH2 activation may be regarded as a new prospective for neuropsychiatric diseases related to brain 5-HT levels. *TPH2* rs4290270 A>T polymorphism was related to the efficacy of disulfiram treatment for cocaine addiction. Carriers of the *TPH2* rs4290270 A allele may respond more favourably to disulfiram than a placebo[51]. Conversely, haplotypes of *TPH2* (rs4570625, rs7963720, rs4760816, rs7305115, rs4290270, and rs17110747) were associated with heroin addiction[24]. In the present study, *TPH2* rs9325202 G>A and rs 1487275 T>G polymorphisms were demonstrated to be associated with performance of domain of Physical Functioning. The genetic polymorphisms detected in the present study are not the same as those identified in other studies because the minor allele frequencies are influenced by ethnicity. These results support the influence of variants in genes involved in the serotonergic synapse on subject-reported QoL in the MMT population.

Regarding the variants in genes involved in the glutamatergic synapse, *GRIN3A* and *GRM6* were two candidate genes related to heroin addiction and response to MMT, respectively. Glutamate is one of the excitatory neurotransmitters in the human brain, and the glutamatergic projection circuit was discovered to contribute to the development of an addiction[53, 54]. Several genetic variations in the glutamatergic pathway have been reported to be associated with susceptibility to drug addiction[10–12]. The haplotypes of *GRIN3A* rs4807399 C>T and rs2240158 C>T were associated with vulnerability to drug addiction[55]. In terms of *GRM6*, carriers of the AG genotype at rs953741 A>G were found to be at increased risk of being nonresponsive to MMT[23]. However, in the present study, no *GRIN3A* or *GRM6* genetic polymorphism was associated with the QoL of the MMT population. These results imply that glutamatergic synapse alteration may be related to the pathophysiology of mood disorders and addiction, but may not be related to the self-reported QoL of those undergoing MMT.

The strength of the present study is that by adjusting for clinical confounders, the effects of genetic variants on the QoL of the MMT population could be evaluated fairly. Additionally, the participants had been receiving MMT for at least 3 months prior to the study; thus, the results of this study may identify genetic markers affecting the QoL of stable individuals undergoing MMT. However, the present study does have some limitations: the sample size and the characteristics of the participants. Because of the limited sample size, the results should be carefully interpreted when considering the general MMT population. Regarding the characteristics of the participants, we did not enrol patients with HIV infection; thus, we could not detect the influence of HIV infection on the QoL of the MMT population. Furthermore, relapse rate is an indicator of poor QoL. We did not record the relapse rate in the included MMT population; therefore, the associations between genetic variants and relapse rate could not be detected in the present study.

In conclusion, rs 1487275 T>G polymorphism in *TPH2* gene was significantly associated with the domain of Physical functioning of QoL in subjects undergoing MMT. The results of the present study may shed light on the importance of the genetic basis of QoL and provide future directions of clinical MMT practice improvements.

## Supporting information

S1 Table SF-36 measurement model. (PDF)

S2 Table Genotype frequencies and score of each item of SF-36 of the participants. (PDF)

S3 Table Genotypes of *TPH2*, *GRM6*, *GRIN3A* in included participants. (PDF)

S1 Fig LD plot of SNPs in *GRIN3A* from CHB data in 1000 Genome project. (PDF)

S2 Fig LD plot of SNPs in *GRM6* from CHB data in 1000 Genome project. (PDF)

S3 Fig LD plot of SNPs in *TPH2* from CHB data in 1000 Genome project. (PDF)

## References

[1] Karow A, Reimer J, Schafer I, Krausz M, Haasen C, Verthein U. Quality of life under maintenance treatment with heroin versus methadone in patients with opioid dependence. Drug Alcohol Depend. 2010;112(3):209–15. 10.1016/j.drugalcdep.2010.06.009 . 20728288

[2] Barbotte E, Guillemin F, Chau N, Lorhandicap G. Prevalence of impairments, disabilities, handicaps and quality of life in the general population: a review of recent literature. Bull World Health Organ. 2001;79(11):1047–55. ; PubMed Central PMCID: PMCPMC2566690. 11731812 PMC2566690

[3] Yen YF, Yen MY, Su LW, Li LH, Chuang P, Jiang XR, et al Prevalences and associated risk factors of HCV/HIV co-infection and HCV mono-infection among injecting drug users in a methadone maintenance treatment program in Taipei, Taiwan. BMC Public Health. 2012;12:1066 10.1186/1471-2458-12-1066 ; PubMed Central PMCID: PMCPMC3534443. 23227904 PMC3534443

[4] Laudet AB, Becker JB, White WL. Don't wanna go through that madness no more: quality of life satisfaction as predictor of sustained remission from illicit drug misuse. Subst Use Misuse. 2009;44(2):227–52. 10.1080/10826080802714462 ; PubMed Central PMCID: PMCPMC2629650. 19142823 PMC2629650

[5] King AC, Pruitt LA, Phillips W, Oka R, Rodenburg A, Haskell WL. Comparative effects of two physical activity programs on measured and perceived physical functioning and other health-related quality of life outcomes in older adults. J Gerontol A Biol Sci Med Sci. 2000;55(2):M74–83. . 10737689 10.1093/gerona/55.2.m74

[6] Sabouri S, Delavar A, Jabbari H. Quality of life among human immunodeficiency virus-1 infected and human immunodeficiency virus-1/hepatitis C virus co-infected individuals in Iranian patients. Niger Med J. 2016;57(1):49–53. 10.4103/0300-1652.180560 ; PubMed Central PMCID: PMCPMC4859113. 27185979 PMC4859113

[7] Aghayan S, Amiri M, Chaman R, Khosravi A. Quality of Life in Methadone Maintenance Treated Patients in Iran. Int J High Risk Behav Addict. 2015;4(4):e22275 10.5812/ijhrba.22275 ; PubMed Central PMCID: PMCPMC4744900. 26870708 PMC4744900

[8] Lin CY, Chang KC, Wang JD, Lee LJ. Quality of life and its determinants for heroin addicts receiving a methadone maintenance program: Comparison with matched referents from the general population. J Formos Med Assoc. 2016;115(9):714–27. 10.1016/j.jfma.2015.07.007 . 26422442

[9] Yen YF, Chou P, Lin YS, Deng CY. Factors associated with health-related quality of life among injection drug users at methadone clinics in Taipei, Taiwan. J Chin Med Assoc. 2015;78(5):292–8. 10.1016/j.jcma.2015.01.001 . 25732869

[10] Kreek MJ, Zhou Y, Butelman ER, Levran O. Opiate and cocaine addiction: from bench to clinic and back to the bench. Curr Opin Pharmacol. 2009;9(1):74–80. 10.1016/j.coph.2008.12.016 ; PubMed Central PMCID: PMCPMC2741727. 19155191 PMC2741727

[11] Levran O, Peles E, Randesi M, Correa da Rosa J, Ott J, Rotrosen J, et al Glutamatergic and GABAergic susceptibility loci for heroin and cocaine addiction in subjects of African and European ancestry. Prog Neuropsychopharmacol Biol Psychiatry. 2016;64:118–23. 10.1016/j.pnpbp.2015.08.003 ; PubMed Central PMCID: PMCPMC4564302. 26277529 PMC4564302

[12] Nielsen DA, Ji F, Yuferov V, Ho A, He C, Ott J, et al Genome-wide association study identifies genes that may contribute to risk for developing heroin addiction. Psychiatr Genet. 2010;20(5):207–14. 10.1097/YPG.0b013e32833a2106 ; PubMed Central PMCID: PMCPMC3832188. 20520587 PMC3832188

[13] Gass JT, Olive MF. Glutamatergic substrates of drug addiction and alcoholism. Biochem Pharmacol. 2008;75(1):218–65. 10.1016/j.bcp.2007.06.039 ; PubMed Central PMCID: PMCPMC2239014. 17706608 PMC2239014

[14] Leal MB, Michelin K, Souza DO, Elisabetsky E. Ibogaine attenuation of morphine withdrawal in mice: role of glutamate N-methyl-D-aspartate receptors. Prog Neuropsychopharmacol Biol Psychiatry. 2003;27(5):781–5. 10.1016/S0278-5846(03)00109-X . 12921910

[15] Trujillo KA, Akil H. Inhibition of morphine tolerance and dependence by the NMDA receptor antagonist MK-801. Science. 1991;251(4989):85–7. . 1824728 10.1126/science.1824728

[16] Larsen RS, Corlew RJ, Henson MA, Roberts AC, Mishina M, Watanabe M, et al NR3A-containing NMDARs promote neurotransmitter release and spike timing-dependent plasticity. Nat Neurosci. 2011;14(3):338–44. 10.1038/nn.2750 ; PubMed Central PMCID: PMCPMC3474337. 21297630 PMC3474337

[17] Nakanishi N, Tu S, Shin Y, Cui J, Kurokawa T, Zhang D, et al Neuroprotection by the NR3A subunit of the NMDA receptor. J Neurosci. 2009;29(16):5260–5. 10.1523/JNEUROSCI.1067-09.2009 ; PubMed Central PMCID: PMCPMC2703294. 19386922 PMC2703294

[18] Roberts AC, Diez-Garcia J, Rodriguiz RM, Lopez IP, Lujan R, Martinez-Turrillas R, et al Downregulation of NR3A-containing NMDARs is required for synapse maturation and memory consolidation. Neuron. 2009;63(3):342–56. 10.1016/j.neuron.2009.06.016 ; PubMed Central PMCID: PMCPMC3448958. 19679074 PMC3448958

[19] Yuan T, Mameli M, O'Connor EC, Dey PN, Verpelli C, Sala C, et al Expression of cocaine-evoked synaptic plasticity by GluN3A-containing NMDA receptors. Neuron. 2013;80(4):1025–38. 10.1016/j.neuron.2013.07.050 . 24183704

[20] Roozafzoon R, Goodarzi A, Vousooghi N, Sedaghati M, Yaghmaei P, Zarrindast MR. Expression of NMDA receptor subunits in human peripheral blood lymphocytes in opioid addiction. Eur J Pharmacol. 2010;638(1–3):29–32. 10.1016/j.ejphar.2010.04.017 . 20420822

[21] Gallinat J, Gotz T, Kalus P, Bajbouj M, Sander T, Winterer G. Genetic variations of the NR3A subunit of the NMDA receptor modulate prefrontal cerebral activity in humans. J Cogn Neurosci. 2007;19(1):59–68. 10.1162/jocn.2007.19.1.59 . 17214563

[22] Lin YT, Hsieh MH, Liu CC, Hwang TJ, Chien YL, Hwu HG, et al A recently-discovered NMDA receptor gene, GRIN3B, is associated with duration mismatch negativity. Psychiatry Res. 2014;218(3):356–8. 10.1016/j.psychres.2014.04.032 . 24814139

[23] Fonseca F, Gratacos M, Escaramis G, De Cid R, Martin-Santos R, Fernandez-Espejo E, et al Response to methadone maintenance treatment is associated with the MYOCD and GRM6 genes. Mol Diagn Ther. 2010;14(3):171–8. 10.2165/11537650-000000000-00000 . 20560679

[24] Nielsen DA, Barral S, Proudnikov D, Kellogg S, Ho A, Ott J, et al TPH2 and TPH1: association of variants and interactions with heroin addiction. Behav Genet. 2008;38(2):133–50. 10.1007/s10519-007-9187-7 . 18181017

[25] Kalivas PW. The glutamate homeostasis hypothesis of addiction. Nat Rev Neurosci. 2009;10(8):561–72. 10.1038/nrn2515 . 19571793

[26] Lavreysen H, Dautzenberg FM. Therapeutic potential of group III metabotropic glutamate receptors. Curr Med Chem. 2008;15(7):671–84. . 18336281 10.2174/092986708783885246

[27] Kelley AE. Memory and addiction: shared neural circuitry and molecular mechanisms. Neuron. 2004;44(1):161–79. 10.1016/j.neuron.2004.09.016 . 15450168

[28] Nestler EJ. Molecular basis of long-term plasticity underlying addiction. Nat Rev Neurosci. 2001;2(2):119–28. 10.1038/35053570 . 11252991

[29] Cooper JR, Melcer I. The enzymic oxidation of tryptophan to 5-hydroxytryptophan in the biosynthesis of serotonin. J Pharmacol Exp Ther. 1961;132:265–8. . 13695323

[30] Lucki I. The spectrum of behaviors influenced by serotonin. Biol Psychiatry. 1998;44(3):151–62. . 9693387 10.1016/s0006-3223(98)00139-5

[31] Linnoila M, Virkkunen M, Scheinin M, Nuutila A, Rimon R, Goodwin FK. Low cerebrospinal fluid 5-hydroxyindoleacetic acid concentration differentiates impulsive from nonimpulsive violent behavior. Life Sci. 1983;33(26):2609–14. . 6198573 10.1016/0024-3205(83)90344-2

[32] Roy A, Adinoff B, Linnoila M. Acting out hostility in normal volunteers: negative correlation with levels of 5HIAA in cerebrospinal fluid. Psychiatry Res. 1988;24(2):187–94. . 2457227 10.1016/0165-1781(88)90061-3

[33] Nakamura K, Sugawara Y, Sawabe K, Ohashi A, Tsurui H, Xiu Y, et al Late developmental stage-specific role of tryptophan hydroxylase 1 in brain serotonin levels. J Neurosci. 2006;26(2):530–4. 10.1523/JNEUROSCI.1835-05.2006 . 16407550 PMC6674418

[34] Patel PD, Pontrello C, Burke S. Robust and tissue-specific expression of TPH2 versus TPH1 in rat raphe and pineal gland. Biol Psychiatry. 2004;55(4):428–33. 10.1016/j.biopsych.2003.09.002 . 14960297

[35] Zill P, Buttner A, Eisenmenger W, Moller HJ, Ackenheil M, Bondy B. Analysis of tryptophan hydroxylase I and II mRNA expression in the human brain: a post-mortem study. J Psychiatr Res. 2007;41(1–2):168–73. 10.1016/j.jpsychires.2005.05.004 . 16023677

[36] Chung IW, Kim H, Sribney W, Hong JB, Lee CH, Lee KY, et al Tryptophan hydroxylase polymorphism is associated with age of onset of alcoholism related behaviors. Alcohol. 2005;36(1):1–3. 10.1016/j.alcohol.2005.06.004 . 16257348

[37] Nielsen DA, Virkkunen M, Lappalainen J, Eggert M, Brown GL, Long JC, et al A tryptophan hydroxylase gene marker for suicidality and alcoholism. Arch Gen Psychiatry. 1998;55(7):593–602. . 9672049 10.1001/archpsyc.55.7.593

[38] Reuter M, Hennig J. Pleiotropic effect of the TPH A779C polymorphism on nicotine dependence and personality. Am J Med Genet B Neuropsychiatr Genet. 2005;134B(1):20–4. 10.1002/ajmg.b.30153 . 15635702

[39] Barrett JC, Fry B, Maller J, Daly MJ. Haploview: analysis and visualization of LD and haplotype maps. Bioinformatics. 2005;21(2):263–5. 10.1093/bioinformatics/bth457 . 15297300

[40] Machiela MJ, Chanock SJ. LDlink: a web-based application for exploring population-specific haplotype structure and linking correlated alleles of possible functional variants. Bioinformatics. 2015;31(21):3555–7. 10.1093/bioinformatics/btv402 ; PubMed Central PMCID: PMCPMC4626747. 26139635 PMC4626747

[41] Peter Westfall RT, and Wolfinger Russell. Multiple Comparisons and Multiple Tests Using SAS®. Second ed: SAS Institute; 2011.

[42] Tibshirani R. Regression Shrinkage and Selection via the Lasso. Journal of the Royal Statistical Society Series B (Methodological). 1996;58:267–88.

[43] Ming Yuan YL. Model Selection and Estimation in Regression with Grouped Variables. Journal of the Royal Statistical Society, Series B. 2006;68:49–67.

[44] Barsevick A, Frost M, Zwinderman A, Hall P, Halyard M, Consortium G. I'm so tired: biological and genetic mechanisms of cancer-related fatigue. Qual Life Res. 2010;19(10):1419–27. 10.1007/s11136-010-9757-7 ; PubMed Central PMCID: PMCPMC3031957. 20953908 PMC3031957

[45] Ordonana JR, Bartels M, Boomsma DI, Cella D, Mosing M, Oliveira JR, et al Biological pathways and genetic mechanisms involved in social functioning. Qual Life Res. 2013;22(6):1189–200. 10.1007/s11136-012-0277-5 . 23054492

[46] Shi Q, Cleeland CS, Klepstad P, Miaskowski C, Pedersen NL, Gene QOLC. Biological pathways and genetic variables involved in pain. Qual Life Res. 2010;19(10):1407–17. 10.1007/s11136-010-9738-x . 20842532

[47] Sprangers MA, Bartels M, Veenhoven R, Baas F, Martin NG, Mosing M, et al Which patient will feel down, which will be happy? The need to study the genetic disposition of emotional states. Qual Life Res. 2010;19(10):1429–37. 10.1007/s11136-010-9652-2 ; PubMed Central PMCID: PMCPMC2977055. 20419396 PMC2977055

[48] Hampton T. Patients' genes may influence quality of life after cancer chemotherapy. JAMA. 2004;292(6):673–4. 10.1001/jama.292.6.673 . 15304448

[49] Sloan JA, Zhao CX. Genetics and quality of life. Curr Probl Cancer. 2006;30(6):255–60. 10.1016/j.currproblcancer.2006.09.001 . 17123877

[50] Lazary J, Viczena V, Dome P, Chase D, Juhasz G, Bagdy G. Hopelessness, a potential endophenotpye for suicidal behavior, is influenced by TPH2 gene variants. Prog Neuropsychopharmacol Biol Psychiatry. 2012;36(1):155–60. 10.1016/j.pnpbp.2011.09.001 . 21946031

[51] Nielsen DA, Harding MJ, Hamon SC, Huang W, Kosten TR. Modifying the role of serotonergic 5-HTTLPR and TPH2 variants on disulfiram treatment of cocaine addiction: a preliminary study. Genes Brain Behav. 2012;11(8):1001–8. 10.1111/j.1601-183X.2012.00839.x ; PubMed Central PMCID: PMCPMC3521860. 22925276 PMC3521860

[52] Matthes S, Mosienko V, Bashammakh S, Alenina N, Bader M. Tryptophan hydroxylase as novel target for the treatment of depressive disorders. Pharmacology. 2010;85(2):95–109. 10.1159/000279322 . 20130443

[53] D'Souza MS. Glutamatergic transmission in drug reward: implications for drug addiction. Front Neurosci. 2015;9:404 10.3389/fnins.2015.00404 ; PubMed Central PMCID: PMCPMC4633516. 26594139 PMC4633516

[54] Nestler EJ. Is there a common molecular pathway for addiction? Nat Neurosci. 2005;8(11):1445–9. 10.1038/nn1578 . 16251986

[55] Xie X, Liu H, Zhang J, Chen W, Zhuang D, Duan S, et al Association between genetic variations of NMDA receptor NR3 subfamily genes and heroin addiction in male Han Chinese. Neurosci Lett. 2016;631:122–5. 10.1016/j.neulet.2016.08.025 . 27542340

