# Supplementary material for: Impacts of GRIN3A, GRM6 and TPH2 genetic polymorphisms on quality of life in methadone maintenance therapy population
Source: PLoS One. 2018 Jul 30;13(7):e0201408. doi: 10.1371/journal.pone.0201408 (PMC6066242; doi:10.1371/journal.pone.0201408)
Supplement: S1 Table — (PDF) [file pone.0201408.s001.pdf]

S1 Table. SF-36 measurement model.

Items	Scales	Summary measures
Vigorous activities	Physical functioning	Physical health
Moderate activities		
Lifting or carrying groceries		
Climb several flights		
Climb one flight		
Bending, kneeling or stooping		
Walk mile		
Walk several blocks		
Walk one block		
Bathing or dressing yourself		
Cut down time	Role-physical	
Accomplished less		
Limited in kind		
Had difficulty		
Pain-magnitude	Bodily pain	
Pain-interfere		
EVGFP rating	General health	
Sick easier		
As healthy		
Health to get worse		
Health excellent		
Pep life	Vitality	Mental health
Energy		
Worn out		
Tired		
Social-extent	Social functioning	
Social-time		
Cut down time	Role-emotional	
Accomplished less		
Not careful		
Nervous	Mental health	
Down in dumps		
Peaceful		
Blue/sad		
Happy		
