# Supplementary material for: Impacts of GRIN3A, GRM6 and TPH2 genetic polymorphisms on quality of life in methadone maintenance therapy population
Source: PLoS One. 2018 Jul 30;13(7):e0201408. doi: 10.1371/journal.pone.0201408 (PMC6066242; doi:10.1371/journal.pone.0201408)
Supplement: S2 Table — (PDF) [file pone.0201408.s002.pdf]

S2 Table. Genotype frequencies and score of each item of SF-36 of the participants.

Gene	SNP No.	Genotypes	Physical Functioning		Role-Physical		Bodily Pain		General Health		Vitality		Social Functioning		Role-Emotional		Mental Health		Healthy	
			N	Mean±SD	N	Mean±SD	N	Mean±SD	N	Mean±SD	N	Mean±SD	N	Mean±SD	N	Mean±SD	N	Mean±SD	N	Mean±SD
GRIN3A	rs7030238	AA	140	24.48±4.78	140	5.41±1.58	142	9.13±1.62	140	13.76±3.33	140	13.37±2.79	141	6.55±1.50	140	4.01±1.25	140	17.71±3.18	142	3.85±1.09
		CA	88	24.70±4.86	87	5.33±1.60	88	9.23±1.92	88	14.51±3.88	87	13.60±3.33	87	6.56±1.45	87	4.03±1.28	87	17.98±3.84	89	3.66±1.02
		CC	15	23.47±5.19	15	5.13±1.55	16	8.51±2.14	15	13.53±3.35	15	13.13±3.62	16	6.38±1.50	15	3.60±0.99	15	16.40±4.34	16	3.25±1.48
	rs1983812	GG	116	24.75±4.74	117	5.60±1.68	119	8.99±2.02	116	14.39±3.86	115	13.80±3.26	118	6.58±1.54	117	4.09±1.27	115	18.17±3.85	119	3.67±1.15
		GA	107	24.31±4.81	105	5.25±1.48	106	9.09±1.73	106	14.39±3.86	106	13.15±2.60	105	6.46±1.42	105	3.98±1.23	106	17.35±2.95	107	3.79±1.00
		AA	30	24.37±5.31	30	4.87±1.38	31	9.22±1.70	31	13.26±3.44	30	13.27±3.41	31	6.42±1.67	30	3.63±1.16	30	17.33±3.82	31	3.87±1.20
	rs942142	AA	32	24.03±4.73[a]	32	5.06±1.29[d]	32	9.17±1.46	32	14.18±2.96	32	13.13±3.05	32	6.75±1.27	32	3.94±1.16	32	17.44±3.53	32	3.81±1.15
		CA	77	24.79±4.91	78	5.28±1.59	79	8.89±1.81	78	13.81±3.51	76	13.51±3.13	78	6.35±1.53	78	4.00±1.28	76	17.47±3.27	79	3.82±1.09
		CC	10	27.00±4.08	10	6.40±1.58	10	8.95±1.52	9	13.91±4.13	10	12.40±2.32	10	7.00±1.63	10	4.60±1.26	10	18.70±3.02	10	4.20±0.79
	rs10512285	AA	169	24.33±4.72	167	5.34±1.56	170	9.14±1.90	169	14.17±3.54	168	13.44±3.01	169	6.54±1.50	167	3.95±1.22	168	17.79±3.63	171	3.71±1.09
		AG	69	24.90±4.93	70	5.27±1.60	71	8.97±1.67	70	13.79±3.61	68	13.53±3.15	70	6.33±1.53	70	4.00±1.30	68	17.29±3.28	71	3.79±1.12
		GG	12	27.00±3.79	12	6.42±1.56	12	9.23±1.63	11	13.93±3.81	12	12.92±2.64	12	7.17±1.59	12	4.67±1.23	12	19.25±3.14	12	3.92±1.16
	rs3983721	CC	100	24.94±4.50	99	5.45±1.69	101	9.21±1.70	98	14.02±3.97	98	13.82±3.53	100	6.51±1.55	99	4.02±1.29	98	17.77±3.80	101	3.69±1.15
		CT	101	24.39±5.05	101	5.46±1.51	103	9.10±1.91	103	14.44±3.21	101	13.37±2.30	102	6.65±1.40	101	4.08±1.25	101	17.81±3.02	104	3.63±1.12
		TT	47	24.17±4.80	47	4.98±1.45	47	8.85±1.92	47	13.23±3.42	47	12.81±3.27	47	6.23±1.66	47	3.74±1.13	47	17.57±3.99	47	4.06±0.87
GRM6	rs17078853	TT	186	24.40±4.81	186	5.25±1.52[e]	189	9.11±1.84	185	14.00±3.58	184	13.49±3.14	187	6.52±1.49	186	3.92±1.21	184	17.80±3.63	189	3.75±1.09
		GT	59	25.12±4.69	58	5.81±1.75	59	9.02±1.80	60	14.29±3.41	59	13.49±2.67	59	6.53±1.62	58	4.21±1.33	59	17.64±3.22	60	3.77±1.08
		GG	7	23.43±6.43	7	4.86±1.21	7	8.07±2.92	7	13.97±3.69	7	12.57±3.05	7	6.00±0.82	7	3.71±1.11	7	16.29±2.50	7	3.29±1.25
	rs2071247	GG	88	24.32±4.59	89	5.28±1.48	89	9.08±1.86	87	13.59±3.53	87	13.44±2.90	87	6.45±1.50	89	3.91±1.21	87	17.78±3.48	89	3.67±1.16
		AG	120	24.53±4.87	118	5.38±1.62	121	9.13±1.81	120	14.56±3.65	119	13.59±3.09	121	6.53±1.53	118	4.11±1.29	119	17.86±3.27	122	3.81±1.06
		AA	42	24.95±5.29	42	5.55±1.68	43	8.90±2.08	43	13.55±3.14	42	13.48±2.88	43	6.58±1.45	42	3.83±1.21	42	17.43±4.10	43	3.72±1.10
	rs17078877	AA	183	24.45±4.72	183	5.26±1.52	186	9.12±1.85	182	13.95±3.60	184	13.46±3.14	181	6.52±1.50	183	3.92±1.22	181	17.81±3.66	186	3.77±1.08
		GA	58	25.29±4.54	57	5.77±1.77	58	9.02±1.82	59	14.29±3.44	58	13.43±2.65	58	6.52±1.64	57	4.19±1.34	58	17.62±3.23	59	3.76±1.09
		GG	7	23.43±6.43	7	4.86±1.21	7	8.07±2.92	7	13.97±3.69	7	12.57±3.05	7	6.00±0.82	7	3.71±1.11	7	16.29±2.50	7	3.29±1.25
	rs11746675	CC	100	24.27±5.11	99	5.12±1.50[f]	100	9.02±1.88	100	14.21±3.81	99	13.38±3.23	100	6.45±1.52	99	3.93±1.24	99	17.33±3.77	100	3.85±1.04
		CT	116	24.69±4.48	116	5.63±1.62	118	9.21±1.69	115	14.17±3.52	115	13.74±2.98	116	6.68±1.43	116	4.07±1.24	115	18.10±3.39	119	3.71±1.06
		TT	32	24.84±5.21	32	5.31±1.62	33	8.74±2.45	33	13.28±2.90	32	12.69±2.56	33	6.24±1.66	32	4.03±1.33	32	17.72±3.15	33	3.52±1.40
	rs2067011	CC	108	24.34±5.08	106	5.22±1.52	108	9.05±1.93	108	14.47±3.63	106	13.63±3.11	107	6.54±1.46	106	4.01±1.26	106	17.73±3.82	109	3.88±1.01
		CT	34	24.15±3.98	35	5.51±1.60	35	9.13±1.34	35	13.73±3.46	35	13.63±3.25	35	6.69±1.59	35	4.00±1.16	35	17.29±3.48	35	3.54±1.07
		TT	30	24.73±5.34	30	5.03±1.47	32	8.89±1.79	31	13.52±2.95	30	12.97±2.22	32	6.22±1.36	30	3.90±1.30	30	17.30±2.47	32	3.59±1.29
TPH2	rs2129575	GG	63	24.32±4.28	63	5.37±1.64	65	9.36±1.73	63	14.09±4.07	61	13.92±3.06	63	6.68±1.64	63	4.02±1.30	61	18.30±3.57	65	3.72±1.21
		GT	126	24.06±5.09	125	5.31±1.46	125	8.92±1.87	125	13.83±3.51	126	13.11±3.15	125	6.39±1.48	125	3.97±1.19	126	17.27±3.55	126	3.74±1.13
		TT	63	24.59±4.79	63	5.46±1.75	65	9.04±1.99	64	14.37±3.05	63	13.73±2.68	65	6.54±1.42	63	3.98±1.31	63	18.05±3.29	65	3.78±1.13
	rs1386493	CC	169	24.47±4.92	168	5.37±1.56	170	9.14±1.76	168	13.91±3.50	168	13.36±2.93	169	6.54±1.50	168	4.04±1.26	168	17.88±3.29	171	3.74±1.14
		CT	70	25.30±4.55	70	5.47±1.68	72	9.05±2.02	71	14.37±3.50	69	13.77±3.25	71	6.52±1.53	70	3.93±1.23	69	17.55±4.07	72	3.69±1.04
		TT	9	24.56±3.50	9	4.67±1.32	9	8.83±1.57	9	13.69±3.25	9	12.56±3.32	9	5.89±1.62	9	3.67±1.32	9	16.44±3.50	9	4.11±0.78
	rs2171363	TT	68	23.90±5.02	68	5.26±1.61	70	9.12±1.75	69	14.36±2.87	68	13.62±2.60	70	6.46±1.59	68	4.10±1.29	68	17.81±3.45	70	3.74±0.88
		TC	125	25.00±4.67	125	5.44±1.56	127	9.06±1.86	125	13.91±3.79	125	13.36±3.22	126	6.50±1.50	125	3.96±1.21	125	17.53±3.54	127	3.72±1.19
		CC	54	24.52±4.42	53	5.38±1.60	53	9.17±1.88	53	13.75±3.86	52	13.33±3.04	52	6.50±1.50	53	3.96±1.29	52	18.04±3.61	54	3.93±1.10
	rs7305115	AA	65	23.83±5.10	65	5.17±1.57	68	8.85±1.85	67	14.27±2.87	65	13.40±2.62	68	6.37±1.56	65	3.98±1.23	65	17.55±3.23	68	3.75±0.89
		AG	130	24.94±4.80	130	5.46±1.58	131	9.11±1.88	129	14.03±3.79	130	13.48±3.22	130	6.61±1.49	130	4.01±1.24	130	17.65±3.21	131	3.69±1.20
		GG	55	24.45±4.47	54	5.39±1.63	54	9.24±1.89	54	13.74±3.80	53	13.45±3.07	53	6.45±1.49	54	3.96±1.30	53	18.15±3.64	55	3.89±1.10
	rs10506645	CC	109	24.95±4.20	108	5.38±1.63	109	9.22±1.89	108	14.03±3.94	106	13.73±3.32	107	6.49±1.51	108	3.94±1.28	106	17.92±3.95	110	3.78±1.11
		TC	106	24.63±5.23	106	5.48±1.56	108	8.90±1.90	107	13.85±3.37	107	13.12±2.79	108	6.51±1.51	106	4.04±1.23	107	17.44±3.31	108	3.78±1.14
		TT	34	22.97±5.13	34	5.03±1.49	35	9.05±1.75	34	14.61±2.87	34	13.76±2.75	35	6.54±1.60	34	4.06±1.23	34	18.06±2.73	35	3.51±0.92
	rs4760820	CC	204	24.49±4.82	203	5.37±1.59	207	9.05±1.80	206	14.13±3.50	203	13.51±3.12	206	6.53±1.55	203	4.00±1.24	203	17.71±3.58	208	3.71±1.09
		CG	46	24.57±4.97	46	5.28±1.50	46	9.09±2.20	44	13.61±3.85	45	13.27±2.66	45	6.42±1.34	46	3.913±1.26	45	17.76±3.32	46	3.89±1.14
		GG	2	25.00±4.24	2	7.50±0.71	2	9.80±0.85	2	14.50±2.12	2	13.00±2.83	2	6.50±0.71	2	4.50±2.12	2	18.06±2.73	2	3.50±0.71
	rs9325202	GG	87	25.14±4.12[b]	88	5.42±1.62	88	9.05±1.97	87	14.26±3.53	86	13.59±3.08	87	6.63±1.32	88	4.01±1.25	86	18.00±3.76	88	3.66±1.18
		AG	117	24.70±5.22	115	5.49±1.59	118	9.07±1.71	117	13.81±3.76	116	13.28±3.05	117	6.42±1.64	115	3.97±1.25	116	17.55±3.36	119	3.84±1.09
		AA	45	22.82±4.57	45	4.91±1.41	46	9.27±1.83	45	14.33±3.05	45	13.80±2.81	46	6.52±1.56	45	3.96±1.26	45	17.76±3.49	46	3.63±0.93
	rs1487275	TT	105	25.16±4.35[c]	106	5.56±1.65	107	9.01±2.01	105	14.09±3.60	104	13.41±3.06	106	6.55±1.45	106	4.02±1.26	104	17.91±3.55	107	3.71±1.11
		GT	119	24.39±4.85	117	5.26±1.52	120	9.11±1.68	119	14.09±3.59	118	13.52±3.10	119	6.58±1.51	117	3.97±1.22	118	17.69±3.56	121	3.79±1.09
		GG	25	22.52±6.00	25	4.96±1.51	25	9.12±2.22	25	13.46±3.40	25	13.32±2.70	25	6.00±1.73	25	3.96±1.37	25	17.00±3.37	25	3.68±1.11

Data filled with gray denoted $P<0.05$ as compared among genotypes. After Bonferroni corrections for multiple comparisons (significance level: $P=0.05/18=0.002$), none reached significantly level. Each genotype tested for SF items with One-way ANOVA.

[a], P=0.03143 ; [b], P=0.0090 ; [c], 0.03339 ; [d], P=0.03049 ; [e], P=0.03987 ; [f], P=0.03122.
