# Supplementary material for: Impacts of GRIN3A, GRM6 and TPH2 genetic polymorphisms on quality of life in methadone maintenance therapy population
Source: PLoS One. 2018 Jul 30;13(7):e0201408. doi: 10.1371/journal.pone.0201408 (PMC6066242; doi:10.1371/journal.pone.0201408)
Supplement: S3 Table — (PDF) [file pone.0201408.s003.pdf]

S3 Table. Genotypes of *TPH2*, *GRM6*, *GRIN3A* in included participants.

Gene in Human Genome build	Chromosome	Variant ID	Variant	Chromosome location	Location in gene	Genotype	Genotype N	Allele	Allele freq.
GRIN3A	9	rs7030238	A>C	101570213	3' UTR	AA	149	A	0.76
(GRCh38						CA	91	C	0.24
.p12)						CC	16		
		rs1983812	G>A	101570761	3' UTR	GG	124	G	0.67
						GA	111	A	0.33
						AA	32		
		rs942142	A>C	101670591	Exon 3	AA	32	A	0.59
					(Ala > Ala)	CA	83	C	0.41
						CC	10		
		rs10512285	A>G.	101670752	Exon 3	AA	177	A	0.81
					(Leu > Leu)	AG	75	G	0.19
						GG	12		
		rs3983721	C>T	101733129	Intron 1	CC	102	C	0.60
						CT	111	T	0.40
						TT	49		
GRM6	5	rs17078853	T>G	178981601	3' UTR	TT	196	T	0.86
(GRCh38						GT	63	G	0.14
.p12)						GG	7		
		rs2071247	G>A	178983150	Exon 9	GG	92	G	0.59
					(Thr > Thr)	AG	127	A	0.41
						AA	45		
		rs17078877	A>G	178983212	Exon 9	AA	193	A	0.85
					(Met > Val)	GA	62	G	0.15
						GG	7		
		rs11746675	C>T	178986946	Exon 7	CC	103	C	0.63
					(Gly > Gly)	CT	122	T	0.37
						TT	37		
		rs2067011	C>T	178988936	Exon 6	CC	111	C	0.71
					(Asn >	CT	35	T	0.29
					Asn)	TT	36		
TPH2	12	rs2129575	T>G	71946293	Intron 4	TT	68	T	0.50
(GRCh38						GT	131	G	0.50
.p12)						GG	67		
		rs1386493	C>T	71961399	Intron 5	CC	179	C	0.82
						CT	74	T	0.18
						TT	9		
		rs2171363	T>C	71966484	Intron 5	TT	73	T	0.53
						TC	131	C	0.47
						CC	57		
		rs7305115	A>G	71979082	Exon 7	AA	71	A	0.52
					(Pro > Pro)	AG	135	G	0.48
						GG	58		
		rs10506645	C>T	71991720	Intron 7	CC	113	C	0.65
					(Pro > Pro)	TC	113	T	0.35
						TT	36		
		rs4760820	C>G	72003216	Intron 8	CC	217	C	0.90
						CG	47	G	0.10
						GG	2		
		rs9325202	G>A	72013697	Intron 8	GG	91	G	0.58
						AG	125	A	0.42
						AA	47		
		rs1487275	T>G	72016512	Intron 8	TT	111	T	0.66
						GT	127	G	0.34
						GG	25		
