# Supplementary material for: Impacts of GRIN3A, GRM6 and TPH2 genetic polymorphisms on quality of life in methadone maintenance therapy population
Source: PLoS One. 2018 Jul 30;13(7):e0201408. doi: 10.1371/journal.pone.0201408 (PMC6066242; doi:10.1371/journal.pone.0201408)
Supplement: S1 Fig — (PDF) [file pone.0201408.s004.pdf]

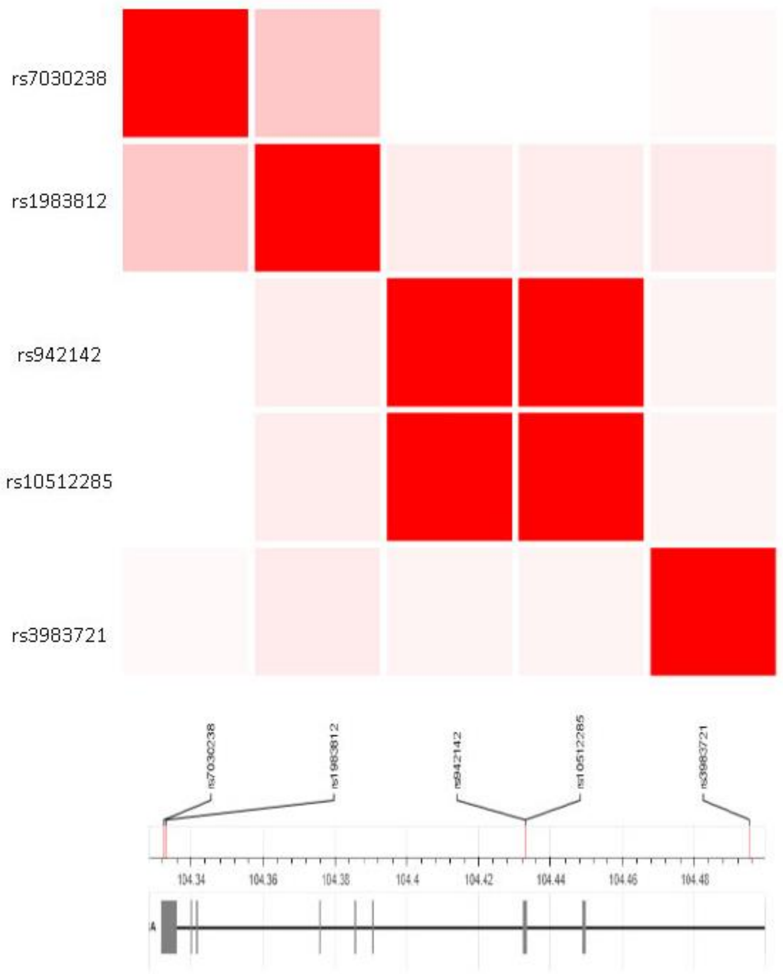

R^2 value for *GRIN3A* gene

rs_number	rs7030238	rs1983812	rs942142	rs10512285	rs3983721
rs7030238	1.0	0.217	0.001	0.001	0.029
rs1983812	0.217	1.0	0.075	0.075	0.083
rs942142	0.001	0.075	1.0	1.0	0.045
rs10512285	0.001	0.075	1.0	1.0	0.045
rs3983721	0.029	0.083	0.045	0.045	1.0

S1 Figure. **LD plot of SNPs in *GRIN3A* from CHB data in 1000 Genome project.** Genomic locations of the genetic polymorphisms on chromosome 9. LDlink website (https://analysistools.nci.nih.gov/LDlink/?tab=home) was used to estimate the linkage disequilibrium blocks. The R^2 values were shown in the figure; red indicated strong linkage disequilibrium.
