# Supplementary material for: Impacts of GRIN3A, GRM6 and TPH2 genetic polymorphisms on quality of life in methadone maintenance therapy population
Source: PLoS One. 2018 Jul 30;13(7):e0201408. doi: 10.1371/journal.pone.0201408 (PMC6066242; doi:10.1371/journal.pone.0201408)
Supplement: S2 Fig — (PDF) [file pone.0201408.s005.pdf]

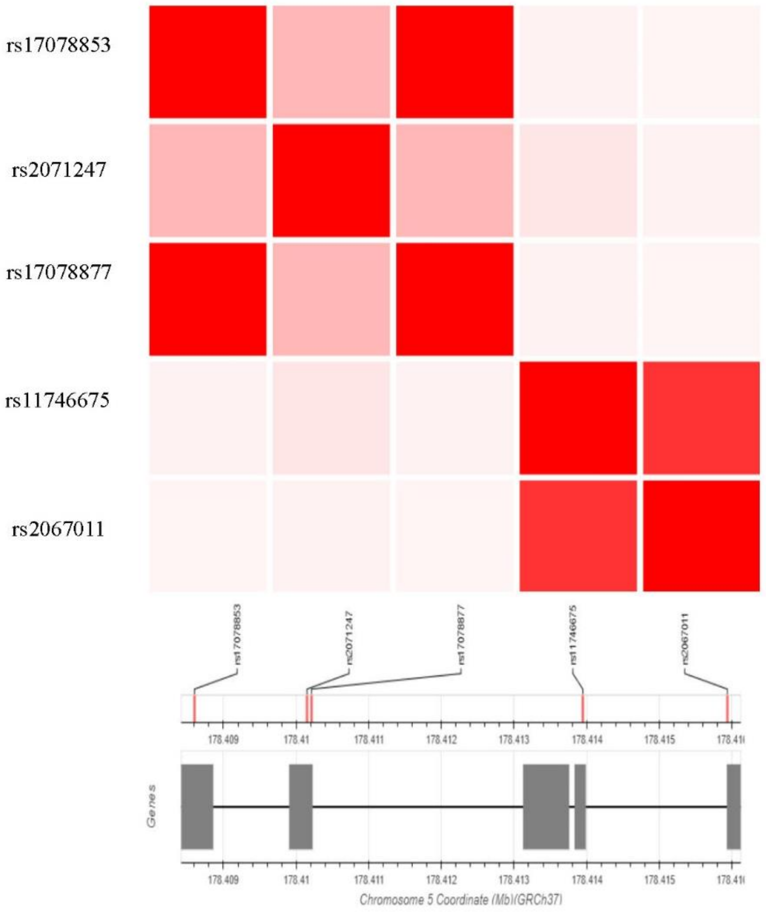


$R^2$ value for *GRM6* gene

| rs_number | rs17078853 | rs2071247 | rs17078877 | rs11746675 | rs2067011 |
|---|---|---|---|---|---|
| rs17078853 | 1.0 | 0.287 | 1.0 | 0.056 | 0.043 |
| rs2071247 | 0.287 | 1.0 | 0.287 | 0.103 | 0.056 |
| rs17078877 | 1.0 | 0.287 | 1.0 | 0.056 | 0.043 |
| rs11746675 | 0.056 | 0.103 | 0.056 | 1.0 | 0.802 |
| rs2067011 | 0.043 | 0.056 | 0.043 | 0.802 | 1.0 |

S2 Figure. **LD plot of SNPs in *GRM6* from CHB data in 1000 Genome project.** Genomic locations of the genetic polymorphisms on chromosome 5. LDlink website (https://analysistools.nci.nih.gov/LDlink/?tab=home) was used to estimate the linkage disequilibrium blocks. The $R^2$ values were shown in the figure; red indicated strong linkage disequilibrium.
