# Supplementary material for: Impacts of GRIN3A, GRM6 and TPH2 genetic polymorphisms on quality of life in methadone maintenance therapy population
Source: PLoS One. 2018 Jul 30;13(7):e0201408. doi: 10.1371/journal.pone.0201408 (PMC6066242; doi:10.1371/journal.pone.0201408)
Supplement: S3 Fig — (PDF) [file pone.0201408.s006.pdf]

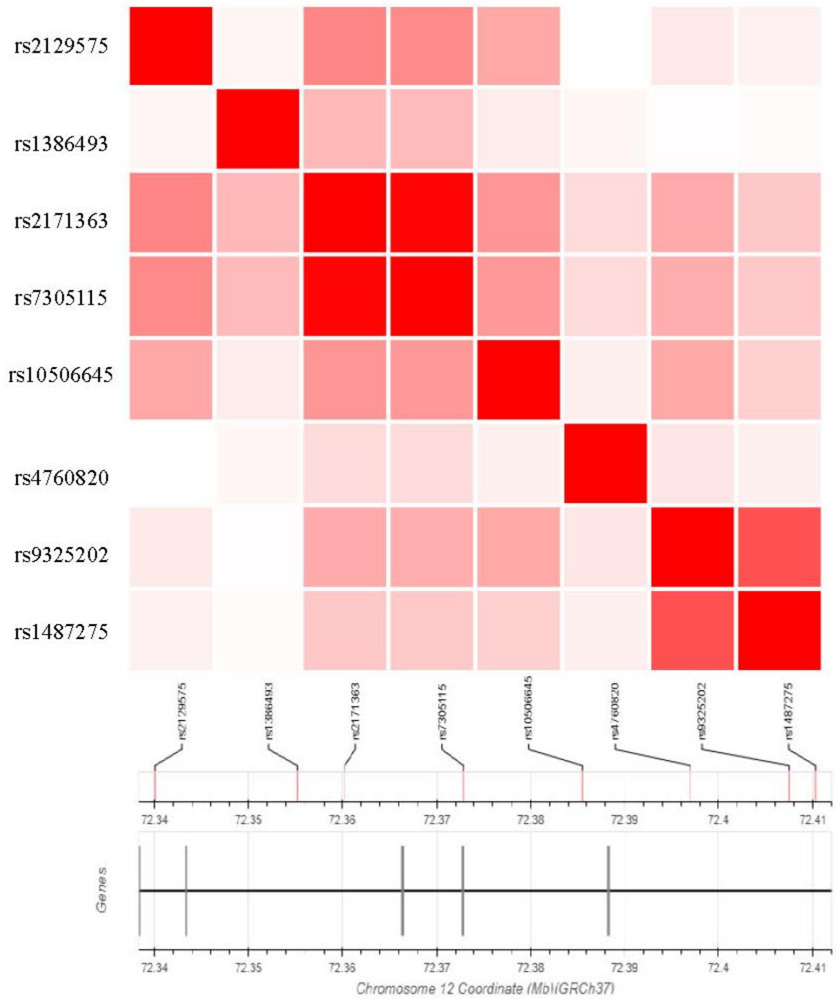

R^2 value for *TPH2* gene

rs_number	rs2129575	rs1386493	rs2171363	rs7305115	rs10506645	rs4760820	rs9325202	rs9325202
rs2129575	1.0	0.044	0.475	0.462	0.343	0.0	0.085	0.059
rs1386493	0.044	1.0	0.277	0.271	0.074	0.039	0.01	0.02
rs2171363	0.475	0.277	1.0	0.981	0.416	0.14	0.333	0.22
rs7305115	0.462	0.271	0.981	1.0	0.408	0.142	0.323	0.213
rs10506645	0.343	0.074	0.416	0.408	1.0	0.062	0.341	0.186
rs4760820	0.0	0.039	0.14	0.142	0.062	1.0	0.102	0.063
rs9325202	0.085	0.01	0.333	0.323	0.341	0.102	1.0	0.685
rs1487275	0.059	0.02	0.22	0.213	0.186	0.063	0.685	1.0

S3 Figure. **LD plot of SNPs in *TPH2* from CHB data in 1000 Genome project.** Genomic locations of the genetic polymorphisms on chromosome 12. LDlink website (https://analysistools.nci.nih.gov/LDlink/?tab=home) was used to estimate the linkage disequilibrium blocks. The R^2 values were shown in the figure; red indicated strong linkage disequilibrium.
